# Triglyceride-glucose index and the outcome of peritoneal dialysis patients: analysis of two cohorts

**DOI:** 10.1186/s12882-026-05110-w

**Published:** 2026-06-10

**Authors:** Miłosz Miedziaszczyk, Jack Kit-Chung Ng, Winston Wing-Shing Fung, Gordon Chun-Kau Chan, Kai-Ming Chow, Cheuk-Chun Szeto

**Affiliations:** 1 Department of General and Transplant Surgery, Poznan University of Medical Sciences, Poznan, Poland; 2 Department of Clinical Pharmacy and Biopharmacy, Poznan University of Medical Sciences, Poznan, Poland; 3 Carol & Richard Yu Peritoneal Dialysis Research Centre, Prince of Wales Hospital, Department of Medicine & Therapeutics, The Chinese University of Hong Kong, Shatin, Hong Kong China; 4 Li Ka Shing Institute of Health Sciences (LiHS), Faculty of Medicine, The Chinese University of Hong Kong, Shatin, Hong Kong China

**Keywords:** Obesity, Metabolic syndrome, Frailty, Renal failure

## Abstract

**Background:**

Cardiovascular disease is the leading cause of death in patients with chronic kidney disease and those on peritoneal dialysis (PD), with insulin resistance contributing significantly to adverse outcomes. This study evaluates the triglyceride-glucose (TyG) index as a prognostic marker for metabolic dysfunction in PD patients.

**Methods:**

We studied two patient cohorts: 278 new PD patients and 178 prevalent ones. TyG index values were calculated by a validated formula. The patients were followed for 28.0 ± 12.6 (incident PD cohort) and 43.9 ± 37.6 months (prevalent PD cohort) for survival and hospitalization.

**Results:**

The mean TyG index was 8.70 ± 0.61 and 9.01 ± 0.72 in the new and prevalent PD cohort, respectively (*p* < 0.0001). In the incident PD cohort, the 2-year patient survival rates for quartiles I to IV of TyG were 84.0%, 85.9%, 79.8%, and 73.8%, respectively (*p* = 0.045). However, after adjustment for confounders, TyG was not an independent predictor of patient survival (adjusted hazard ratio 0.958, 95% confidence interval 0.732 to 1.123, *p* = 0.753). In the prevalent PD cohort, the 2-year patient survival rates for quartiles I to IV of TyG were 68.9%, 75.0%, 84.4%, and 75.0%, respectively (*p* = 0.364). In both cohorts, TyG index showed no significant association with technique survival rate, hospitalization rates, duration of hospitalization, or peritonitis rates.

**Conclusions:**

The TyG index was higher in prevalent PD patients but was not an independent predictor of patient or technique survival, hospitalization, or peritonitis rate after multivariate adjustment. Although correlated with metabolic parameters, the TyG index did not serve as a prognostic marker in PD patients.

**Supplementary Information:**

The online version contains supplementary material available at 10.1186/s12882-026-05110-w.

## Introduction

Metabolic syndrome is a cluster of conditions—including visceral obesity, hyperglycemia, dyslipidemia, and hypertension—that collectively increase the risk of cardiovascular disease (CVD) and mortality [1, 2]. Patients undergoing dialysis—both hemodialysis (HD) and peritoneal dialysis (PD)—exhibit a high prevalence of metabolic syndrome due to factors such as insulin resistance, chronic inflammation, oxidative stress, and altered lipid metabolism, which are intensified by kidney dysfunction and dialysis-related procedures [3–5]. Additionally, lifestyle factors like reduced physical activity, dietary imbalances, and the presence of comorbidities such as diabetes and hypertension further contribute to its development. These elements significantly elevate their cardiovascular risk [2–4, 6].

Metabolic syndrome is common in PD patients and may be associated with cardiovascular disease risk. In addition, PD-related metabolic effects, such as weight gain, dyslipidaemia and impaired glucose tolerance or new-onset diabetes, can exacerbate its components. Traditionally, metabolic syndrome is diagnosed using established criteria such as those from the World Health Organization (WHO) [7] or the National Cholesterol Education Program (NCEP) Expert Panel (Adult Treatment Panel III, ATP III) [8]. However, these criteria require comprehensive assessments of various metabolic parameters and provide a binary classification—either presence or absence of syndrome. In this context, the triglyceride-glucose (TyG) index, calculated from fasting triglyceride and glucose levels, has emerged as a promising marker for insulin resistance and metabolic syndrome [9, 10].

The prognostic significance of the TyG index has been evaluated in multiple studies. In the general population, a higher TyG index is associated with an increased risk of cardiovascular events and mortality [11–13]. Among HD patients, an elevated baseline TyG independently predicts cardiovascular mortality [14, 15] and all-cause mortality [15]. For dialysis patients with coronary artery disease, the TyG index correlates with the severity of coronary atherosclerosis and mortality [16].

Despite the high prevalence of metabolic disturbances in PD patients, research on the prognostic value of the TyG index in this group remains limited [17–19]. Our previous work demonstrated that changes in the TyG index track with alterations in insulin resistance among PD patients [20], suggesting that the TyG index is a valuable surrogate marker of metabolic dysfunction. The aim of this study is to assess the role of the TyG index as a prognostic indicator for PD patients.

## Patients and methods

The study received approval from the Joint Chinese University Hong Kong-New Territories East Cluster Clinical Research Ethics Committee (approval number CRE-2015.250 and CRE-2021.367). All procedures adhered to the guidelines outlined in the Declaration of Helsinki.

### Study design

This is a retrospective analysis of two prospective observational studies conducted at a single center in a tertiary academic hospital. The incident PD cohort recruited consecutive adult patients started on PD from January 2021 to June 2023; the prevalent PD cohort recruited existing PD patients from April 2008 to August 2009. After written informed consent, we performed routine anthropometric measurements, blood biochemistry after 8-hour overnight fasting, multi-frequent bioimpedance spectroscopy, arterial pulse wave velocity study, dialysis small solute clearance, and other nutritional assessment. Charlson’s comorbidity index was computed at the baseline study as previously described [21].

### Triglyceride-glucose index (TyG index)

TyG index was calculated by the following formula as previously described [22].

$${\rm{triglyceride}}\,{\rm{glucose}}\,{\rm{index = ln}}\left[ {{{{\rm{TG}}\left( {{\rm{mg/dL}}} \right){\rm{ \times FG}}\left( {{\rm{mg/dL}}} \right)} \over {\rm{2}}}} \right]$$

### Assessment of peritoneal transport

Standard peritoneal equilibration test (PET) was performed according to the method described by Twardowski [23]. The dialysate-to-plasma creatinine ratio (D/P) was computed after adjusting for the influence of glucose. The mass transfer area coefficients (MTAC) of creatinine normalized for body surface area (BSA) were obtained by applying the standard formula [24]. The BSA was calculated by the formula of Gehan and George [25].

### Small solute clearance and nutritional status

The small solute clearance during dialysis was assessed using the conventional method [26], which involved a 24-hour collection of urine and dialysate to calculate the total Kt/V. The residual glomerular filtration rate (GFR) was estimated by averaging the 24-hour urinary clearances of urea and creatinine [27]. The normalized protein nitrogen appearance (NPNA) was determined using the modified Bergstrom formula [28]. Nutritional status was evaluated through both the Subjective Global Assessment (SGA) overall score [29] and the comprehensive malnutrition-inflammation score (MIS) [30], following previously established methods [31, 32]. Serum albumin levels were measured via the bromocresol purple method. Fat-free, edema-free body mass (FEBM) was calculated using the creatinine kinetic method based on Forbes and Brunning’s formula [33], which requires 24-hour collection of urine and dialysate. FEBM was then expressed as a percentage of the ideal body weight (IBW), calculated from body height and sex using a standard formula validated in Southern Chinese populations [34].

### Bioimpedance spectroscopy

We used the multi-frequency bioimpedance spectroscopy device (Body Composition Monitor, Fresenius Medical Care, Germany) for the assessment of body composition as described previously [5]. In this study, we analyzed the data on lean tissue mass (LTM), adipose tissue mass (ATM), volume of over-hydration (OH), and extracellular-to-intracellular volume (E: I) ratio.

### Arterial pulse wave velocity

Arterial pulse wave velocity (PWV) was measured by an automatic computerized recorder and analyzed using the Complior SP program (Artech Medical, France) by the method described previously [6]. In this study, brachial-ankle, carotid-radial and carotid-femoral PWV were calculated.

### Clinical outcomes

After the baseline assessment, all patients were followed for at least 24 months. During this follow-up period, clinical management was decided by individual physicians and was not influenced by the study parameters. The study outcomes included patient survival, cardiovascular survival, technique survival, peritonitis-free survival, peritonitis rate, number of hospital admission, and duration of hospital stay. For patient survival analysis, recovery of renal function, loss to follow-up, transfer to another dialysis center, conversion to long-term hemodialysis, and kidney transplantation were censored. In the analysis of cardiovascular survival, only deaths caused by cardiac diseases and stroke were counted as events, while deaths of other causes were censored. In the technique survival analysis, patient death, conversion to long-term hemodialysis, and renal transplantation were taken as events, while recovery of renal function, loss to follow-up, and transfers to other centers were censored. The number of hospital admissions and duration of hospital stays were adjusted for the follow-up duration.

### Statistical analysis

Statistical analysis was performed using MedCalc software version 23.1.7 (Ostend, Belgium) and R version 4.4.1 (The R Foundation for Statistical Computing). Missing data, which constituted less than 3% of our dataset, were addressed using multiple imputation by chained equations (MICE). The Shapiro-Wilk test was employed to assess whether the data followed a normal distribution. Demographic and clinical data were compared among quartiles of TyG index by Chi square test or Kruskal Wallis test as appropriate. Correlations between variables were explored by the Spearman’s rank correlation coefficient. Survival rates were depicted by Kaplan–Meier survival curves and analyzed by univariate and multivariate Cox regression between TyG quartiles. In the multivariate Cox regression model for patient survival, we included the Charlson’s score, MIS, serum albumin, and residual GFR into the model because of their well-known prognostic relevance. In addition, a second multivariable Cox model was constructed by screening all available clinical data and include all variables with *p* < 0.1 by the univariate analysis. Hospitalization data and peritonitis rates of individual patient were adjusted for the duration of follow up and compared across TyG quartiles using the Kruskal-Wallis test. A *p*-value of less than 0.05 was considered statistically significant; all probabilities were two-tailed.

## Results

We first studied 278 patients in the new PD cohort and 178 patients in the prevalent PD cohort. The mean TyG index was 8.70 ± 0.61 and 9.01 ± 0.72, respectively (*p* < 0.0001).

### Incident PD cohort

The baseline demographic, clinical, and biochemical characteristics of the incident PD cohort, grouped by the TyG index quartile, are summarized in Tables 1 and 2, respectively. In essence, TyG index was associated with fasting glucose, HbA1C, lipid profile, and adipose tissue mass (Table S1).

**Table 1:** Baseline demographic and clinical characteristics according to TyG index quartile for the incident PD cohort

	All cases	Plasma TyG index quartile				*P***-value**
		I	II	III	IV	
No. of patients	278	70	69	69	70	NA
TyG index	8.70 ± 0.61	7.98 ± 0.29	8.47 ± 0.10	8.83 ± 0.12	9.50 ± 0.39	NA
Age (year)	62.7 ± 12.5	61.8 ± 12.8	62.8 ± 12.3	61.6 ± 14.3	64.4 ± 10.3	0.724 ^a^
Sex, no. of patient (%)						0.356 ^b^
Male	168 (60.4%)	48 (68.6%)	39 (56.5%)	38 (55.1%)	43 (61.4%)	
Female	110 (39.6%)	22 (31.4%)	30 (43.5%)	31 (44.9%)	27 (38.6%)	
Body weight (kg)	66.7 ± 14.3	63.3 ± 13.1	62.0 ± 12.1	65.6 ± 15.0	76.0 ± 15.0	0.153 ^a^
Body mass index (kg/m^2^)	25.2 ± 5.8	23.8 ± 6.3	23.5 ± 3.3	24.4 ± 4.8	29.0 ± 3.1	0.009 ^a^
Blood pressure (mmHg)						
Systolic	142.5 ± 18.6	144.8 ± 18.2	141.5 ± 16.7	142.2 ± 19.4	141.6 ± 20.1	0.774 ^a^
Diastolic	75.7 ± 13.6	79.4 ± 13.4	73.7 ± 13.3	75.7 ± 14.3	74.0 ± 12.8	0.102 ^a^
Primary kidney disease, no. of case (%)						0.004^b^
Diabetes mellitus	150 (54.0%)	29 (41.4%)	35 (50.7%)	32 (46.4%)	54 (77.1%)	
Hypertension	34 (12.2%)	10 (14.3%)	7 (10.1%)	12 (17.4%)	5 (7.1%)	
Glomerulonephritis	54 (19.4%)	14 (20.0%)	17 (24.6%)	16 (23.2%)	7 (2.5%)	
Polycystic kidney disease	10 (3.6%)	2 (2.9%)	4 (5.7%)	3 (4.3%)	1 (1.4%)	
Urological	10 (3.6%)	6 (8.6%)	1 (1.4%)	1 (1.4%)	2 (2.9%)	
Others	7 (2.5%)	3 (4.3%)	0 (0.0%)	3 (4.3%)	1 (1.4%)	
Unknown	13 (4.7%)	6 (8.6%)	5 (7.2%)	2 (2.9%)	0 (0.0%)	
Comorbidities, no. of case (%)						
Diabetes Mellitus	168 (60.4%)	34 (48.6%)	40 (58.0%)	39 (56.5%)	55 (78.6%)	0.002 ^b^
Ischemic heart disease	47 (16.9%)	9 (12.9%)	10 (14.5%)	10 (14.5%)	18 (25.7%)	0.155 ^b^
Cerebrovascular accident	24 (8.6%)	5 (7.1%)	6 (8.7%)	7 (10.1%)	6 (8.6%)	0.941 ^b^
Peripheral vascular disease	11 (4.0%)	1 (1.4%)	3 (4.3%)	4 (5.8%)	3 (4.3%)	0.608 ^b^
Charlson’s comorbidity score	6.0 ± 2.3	5.6 ± 2.2	5.9 ± 2.3	5.9 ± 2.4	6.5 ± 2.2	0.115^b^
PD specific parameters						
APD, no. of case (%)	69 (24.8%)	20 (28.6%)	18 (26.1%)	22 (31.9%)	9 (12.9%)	0.050 ^b^
Icodextrin, no. of case (%)	104 (37.4%)	32 (45.7%)	28 (40.6%)	24 (34.8%)	20 (28.6%)	0.180 ^b^
Exchange volume (L/day)	7.1 ± 1.8	7.0 ± 1.8	7.2 ± 1.6	7.5 ± 2.1	6.6 ± 1.4	0.021^c^
Ultrafiltration volume (L/day)	0.70 ± 0.75	0.80 ± 1.69	0.73 ± 1.59	0.63 ± 1.62	0.63 ± 1.12	0.194^c^
Glucose exposure (g/day)	108.1 ± 27.8	106.5 ± 26.9	111.0 ± 25.7	114.5 ± 32.2	102.0 ± 21.4	0.031^c^

TyG index, triglyceride glucose index; PD, peritoneal dialysis; APD, machine assistance automated peritoneal dialysis.

Data were compared by ^a^Kruskal-Wallis test, ^b^Chi-square test, or ^c^one way analysis of variance (ANOVA).

**Table 2:** Baseline nutrition and biochemical parameters according to plasma TyG index quartile for the incident PD cohort

	All cases	Plasma TyG index quartile				*P***-value**
		I	II	III	IV	
No. of patients	278	70	69	69	70	
Anthropometry						
Waist circumference (cm)	93.4 ± 11.3	90.9 ± 11.2	92.7 ± 11.5	94.5 ± 10.6	95.3 ± 11.6	0.173
Hip circumference (cm)	95.3 ± 10.9	96.0 ± 7.8	93.2 ± 13.6	96.8 ± 8.9	95.2 ± 12.3	0.175
Other nutritional indices						
SGA score	5.5 ± 0.8	5.5 ± 1.0	5.6 ± 0.7	5.6 ± 0.7	5.5 ± 0.8	0.968
MIS score	7.0 ± 3.6	6.8 ± 3.6	6.8 ± 3.5	7.4 ± 3.7	7.2 ± 3.6	0.503
CFS score	3.0 ± 1.2	2.8 ± 0.8	2.9 ± 1.2	3.1 ± 1.3	3.1 ± 1.5	0.649
Hemoglobin (g/dL)	10.51 ± 1.63	10.66 ± 1.65	10.73 ± 1.48	10.36 ± 1.63	10.25 ± 1.75	0.155
Albumin (g/L)	28.01 ± 4.56	28.52 ± 4.53	28.44 ± 4.53	27.33 ± 4.58	27.66 ± 4.62	0.322
Lipid profile (mmol/l)						
Total Cholesterol	4.13 ± 1.12	3.85 ± 1.03	3.76 ± 0.84	4.39 ± 1.32	4.51 ± 1.08	<0.001
Triglyceride	1.48 ± 0.90	0.79 ± 0.18	1.12 ± 0.22	1.55 ± 0.32	2.47 ± 1.20	<0.001
LDL cholesterol	2.19 ± 0.94	1.98 ± 0.76	1.89 ± 0.66	2.50 ± 1.15	2.39 ± 0.97	0.001
HDL cholesterol	1.26 ± 0.47	1.51 ± 0.51	1.35 ± 0.41	1.19 ± 0.41	1.00 ± 0.35	<0.001
Fasting glucose (mmol/L)	6.01 ± 2.02	4.89 ± 0.80	5.60 ± 1.26	5.74 ± 0.97	7.78 ± 2.92	<0.001
HbA1C (%)	6.06 ± 1.01	5.81 ± 1.04	5.92 ± 0.81	5.91 ± 0.75	6.59 ± 1.18	<0.001
Total Kt/V	2.20 ± 0.86	2.22 ± 0.89	2.37 ± 1.13	2.09 ± 0.62	2.11 ± 0.63	0.195
Residual GFR (ml/min/1.73 m^2^)	4.33 ± 2.86	4.23 ± 2.97	4.77 ± 2.96	3.88 ± 2.62	4.40 ± 2.86	0.475
NPNA (g/kg/day)	1.15 ± 0.32	1.18 ± 0.32	1.20 ± 0.43	1.11 ± 0.22	1.12 ± 0.24	0.755
FEBM (kg)	23.37 ± 8.17	24.38 ± 8.69	21.42 ± 5.19	24.17 ± 10.20	23.67 ± 7.83	0.179
Peritoneal transport						
D/P4	0.64 ± 0.29	0.65 ± 0.17	0.68 ± 0.50	0.61 ± 0.15	0.62 ± 0.15	0.636
MTAC (ml/min/1.73 m2)	9.03 ± 5.43	10.12 ± 7.88	8.95 ± 4.32	8.26 ± 3.88	8.80 ± 4.69	0.602
Bioimpedance spectroscopy						
Overhydration (L)	3.99 ± 3.05	4.58 ± 3.35	3.22 ± 2.14	3.91 ± 2.97	4.21 ± 3.47	0.211
E:I ratio	0.99 ± 0.17	0.99 ± 0.17	0.95 ± 0.14	0.99 ± 0.15	1.03 ± 0.21	0.216
Lean tissue mass (kg)	41.71 ± 11.12	43.90 ± 11.00	40.42 ± 9.92	41.97 ± 11.64	40.55 ± 11.69	0.311
Adipose tissue mass (kg)	18.08 ± 10.73	14.58 ± 8.87	17.26 ± 10.32	19.57 ± 12.13	20.83 ± 10.49	0.003
Arterial pulse wave velocity (cm/s)						
Brachial to ankle	13.72 ± 2.19	13.58 ± 2.08	13.53 ± 2.12	13.68 ± 2.26	14.07 ± 2.30	0.530
Carotid-femoral	11.76 ± 7.38	13.40 ± 14.22	11.20 ± 1.99	11.20 ± 1.97	11.23 ± 2.23	0.994
Carotid-radial	10.79 ± 1.66	10.84 ± 1.67	10.84 ± 1.73	10.84 ± 1.52	10.64 ± 1.73	0.811

TyG, triglyceride glucose; SGA, subjective global assessment; MIS, malnutrition inflammation score; CFS, clinical frailty scale; LDL, low-density lipoprotein; HDL, high-density lipoprotein; HbA1C, glycosylated hemoglobin; Kt/V, dialysis adequacy assessment by total urea clearance; GFR, glomerular filtration rate; NPNA, normalized protein nitrogen appearance; FEBM, fat-free edema-free body mass; D/P4, dialysate-to-plasma ratios of creatinine at 4 hours; MTAC, mass transfer area coefficient of creatinine; E: I ratio, extra-cellular to intracellular volume ratio.

Data compared by Kruskal-Wallis test.

**Fig. 1:**
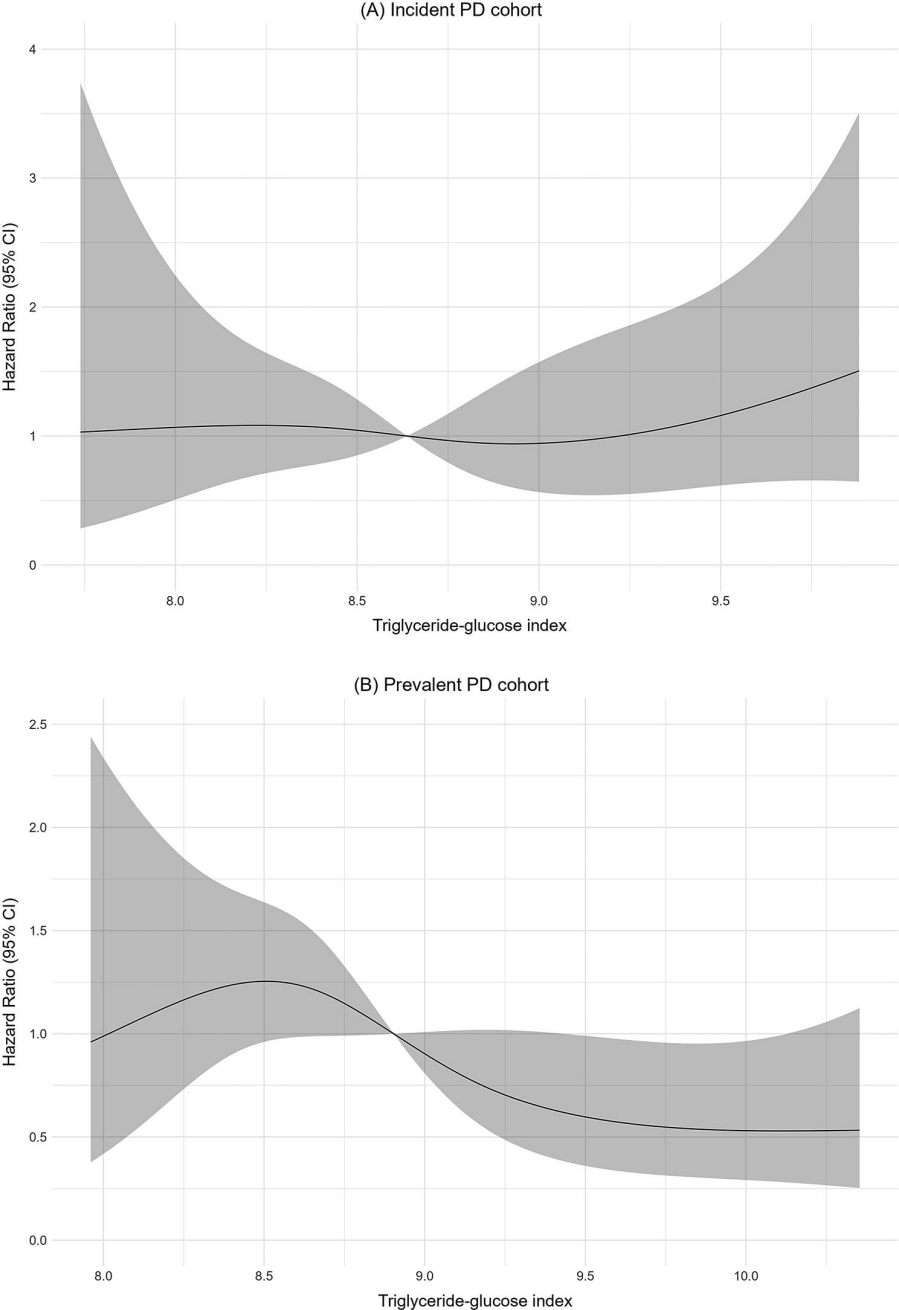
Association between triglyceride-glucose (TyGI) index and patient survival by restricted cubic splines (RCS) analysis: (**A**) incident peritoneal dialysis (PD) cohort; and (**B**) prevalent PD cohort. RCS curves are used to examine the relationship between the TyGI index and the hazard ratio (HR) for survival. The solid line represents the estimated hazard ratio, and the shaded area indicates the 95% confidence interval (CI). The model was adjusted for diabetes status, Charlson comorbidity index, malnutrition-inflammation score (MIS), serum albumin, and residual glomerular filtration rate (GFR), including interaction terms for diabetes status and residual GFR. The reference value (HR = 1.0) was set at the median value of the TyGI index. Knots were placed at the 5th percentiles. For the incident PD cohort, the *p* values for overall association and for non-linearity were 0.219 and 0.083, respectively. For the prevalent PD cohort, the *p* values for overall association and for non-linearity were 0.184 and 0.224, respectively

In this incident PD cohort, the patients were followed for a total of 28.0 ± 12.6 months. During the follow-up period, 62 patients died. The causes of death were ischemic heart diseases (15 patients), cerebrovascular accidents (6 patients), sudden cardiac arrest (8 patients), peritonitis (3 patients), non-peritonitis infection (23 patients), termination of dialysis (4 patients), and other specific causes (3 patients). There was a significant association between TyG quartiles and patient survival by univariate Cox analysis; the 2-year patient survival rates for quartiles I to IV of TyG were 84.0%, 85.9%, 79.8%, and 73.8%, respectively (*p* = 0.045). There was also a trend of association between the absolute TyG value and patient survival, but the result did not reach statistical significance (unadjusted HR 1.370, *p* = 0.103). However, TyG quartile was not an independent predictor of patient survival after adjusting for clinical confounding factors (Table 3). TyG quartiles had no significant association with cardiovascular survival (Table 3). There was no significant interaction between diabetic status and TyG quartile on patient or cardiovascular survival (interaction *p* = 0.667 and *p* = 0.288, respectively). A separate survival model that included all available clinical data also showed no association between TyG quartile and patient survival (Table S2). Restricted cubic spine analysis with TyG as a continuous variable did not show a significant non-linear effect on patient survival (*p* = 0.083) (Fig. 1), or any significant interaction with diabetes status (*p* = 0.598) or residual GFR (*p* = 0.059).

**Table 3:** Cox regression models for patient and cardiovascular survival of the incident PD cohort

Model	Univariate		Multivariate		
Parameter	Unadjusted HR	*P*-value	Adjusted HR	95% Cl	*P*-value
**Patient survival**					
TyG quartile	1.263	0.045	0.997	0.769 to1.291	0.980
Charlson’s comorbidity score	1.203	0.001	1.222	1.080 to 1.384	0.001
MIS	1.128	0.0001	1.061	0.973 to 1.157	0.219
Serum albumin	0.926	0.009	0.957	0.891 to 1.027	0.212
Residual GFR	0.948	0.317	0.995	0.885 to 1.120	0.948
**Cardiovascular survival**					
TyG quartile	1.033	0.856	0.812	0.568 to 1.161	0.304
Charlson’s comorbidity score	1.308	0.0008	1.385	1.167 to 1.643	0.0003
MIS	1.067	0.203	1.052	0.931 to 1.187	0.425
Serum albumin	0.931	0.047	0.936	0.846 to 1.037	0.163
Residual GFR	1.008	0.924	1.067	0.914 to 1.244	0.512

TyG, triglyceride glucose; MIS, malnutrition inflammation score; GFR, glomerular filtration rate.

Data are presented as Hazard ratio (95% confidence interval) and *p* value.

During the study period, 19 patients were switched to long term hemodialysis, 6 had kidney transplantation; and 2 were transferred to other centers. The technique survival rates after 2 years for TyG index quartile I to IV were 76.6%, 72.6%, 72.9%, and 64.8%, respectively (*p* = 0.198).

During the study period, there were 1031 hospital admissions for a total of 9657 days. The overall median rate of hospital admission was 0.95 episodes per year (IQR 0.29 to 2.39), for a median of 4.81 days per year (IQR 0.41 to 22.58). During this period, there were 162 peritonitis episodes; the median rate of peritonitis was 0.00 episode per patient-year (IQR 0.00 to 0.35). The peritonitis-free survival rates after 2 years for TyG quartiles I to IV were 76.5%, 76.4%, 64.6%, and 60.9%, respectively (*p* = 0.144). The relation between the TyG index quartile, hospitalization, and peritonitis rate are summarized in Table S3, and the causative organisms of the peritonitis episodes are summarized in Table S4. In essence, TyG index quartile did not have any significant association with the distribution of causative organism for peritonitis episodes, number of hospital admissions, duration of hospitalization, or hospitalization rate.

### Prevalent PD cohort

There were 178 patients in the prevalent PD cohort. Their baseline demographic, clinical, and biochemical characteristics are summarized in Table S5. The correlation between TyG index and other clinical and biochemical parameters for the prevalent PD cohort was similar to the incident PD cohort (Table S3).

The prevalent PD cohort was followed for 43.9 ± 37.6 months. During the follow-up period, 123 patients died. The causes of death were ischemic heart diseases (31 patients), cerebrovascular accidents (10 patients), sudden cardiac arrest (18 patients), peritonitis (22 patients), non-peritonitis infection (23 patients), termination of dialysis (13 patients), malignancy (3 patients), other specific causes (2 patients), or unknown (1 patient During this period, 30 were switched to long term hemodialysis, 20 had kidney transplantation; and 5 were transferred to other centers. There was no significant association between TyG quartiles and patient survival by univariate Cox analysis; the 2-year patient survival rates for quartiles I to IV of TyG were 68.9%, 75.0%, 84.4%, and 75.0%, respectively (*p* = 0.364). The corresponding 2-year technique survival rates were 57.8%, 56.8%, 75.6%, and 68.2%, respectively (*p* = 0.189). Restricted cubic spine analysis with TyG as a continuous variable also did not show a significant non-linear effect on patient survival (*p* = 0.224) (Fig. 1), or any significant interaction with diabetes status (*p* = 0.058) or residual GFR (*p* = 0.383).

During the follow-up period of the prevalent PD cohort, there were 619 hospital admissions for a total of 4728 days. The overall median rate of hospital admission was 0.59 episodes per year (IQR 0.00 to 1.47), for a median of 3.44 days per year (IQR 0.00 to 9.24). During this period, there were 274 peritonitis episodes; the median rate of peritonitis was 0.23 episode per patient-year (IQR 0.00 to 0.69). The 2-year peritonitis-free survival rates after 2 years for TyG quartiles I to IV were 73.3%, 65.9%, 53.3%, and 65.9%, respectively (*p* = 0.254). In the prevalent PD cohort, TyG index quartile also did not have any significant association with the number of hospital admissions, duration of hospitalization, peritonitis rate, or the distribution of causative organism for the peritonitis episodes (Tables S3 and S4).

## Discussion

In this cohort study, we observed that the TyG index was significantly higher in patients with prevalent PD cohort as compared to the incident PD cohort. While univariate analysis indicated an association between TyG quartiles and patient survival among incident PD cohort, this relationship did not remain significant after multivariate adjustment. Similarly, TyG quartiles were not independently predictive of technique survival, hospitalization rates, or peritonitis-free survival in either cohort. We conclude that although the TyG index correlates with metabolic parameters, it does not serve as an independent prognostic marker for clinical outcomes in PD patients.

CVD is the leading cause of mortality in patients with CKD and, along with infections, remains a major cause of death among those undergoing PD. Increasing evidence suggests that insulin resistance plays a crucial role in these adverse outcomes. Recently, several surrogate markers of insulin resistance, including the TyG index, have emerged as promising tools for risk stratification in various clinical settings [35, 36]. Notably, the TyG index has demonstrated prognostic value in non-dialysis CKD patients, particularly in predicting major cardiovascular events. Multiple studies have established a positive association between higher TyG levels and increased cardiovascular mortality. For instance, among nondiabetic individuals, elevated TyG has been correlated with CKD progression and higher mortality rates [35, 37]. In PD patients, increased TyG levels have been linked to higher cardiovascular mortality, and elevated TyG-BMI has shown a significant association with the risk of cardiovascular death after adjusting for confounders [18]. However, our study found that while higher TyG was associated with reduced survival in univariate analysis, this association lost significance after multivariate adjustments. These discrepancies may be due to differences in patient demographics, treatment protocols, or lower event rates within our cohort. On the other hand, extensive adjustment for clinical parameters in our present study may attenuate the independent prognostic value of TyG.

In this study, we analyzed two highly different cohorts (i.e. incident and prevalent PD patients) recruited during markedly different periods. Although there are possible impact of temporal differences in PD practice, cardiovascular management, glucose exposure, and survival bias in the two cohorts, the results were markedly similar. Similarly, although the prevalent PD cohort is subjected to survivor bias (because patients with the highest metabolic risk may have died before recruitment) and attenuated the association between TyG and clinical outcome, the result is supported by the similar observations in the incident PD cohort. On the other hand, the prevalent PD cohort demonstrated a considerably higher peritonitis rate and worse peritonitis-free survival than the incident PD cohort, probably explained by the gradual change in PD practice over time, with continual improvement in the prevention and treatment of PD related peritonitis. We found no association between TyG index quartiles and peritonitis in either cohort, which contrasts with some prior reports. For example, Liu et al. [19] reported higher PD-related peritonitis rates in patients with elevated TyG, while Wan et al. [38] found that patients in the highest TyG tertile had a markedly increased risk of peritonitis, even after adjustment. Additionally, we observed no significant link between TyG quartiles and technique survival, hospital admission frequency, or length of stay, aligning with previous findings [21]. On the other hand, the sample size of our study was reasonable but may not be sufficient for sophisticated model construction. For the primary analysis with multi-variable Cox model, the event-per-variable ratios of the incident and prevalent PD cohorts were 12.4 and 24.6, respectively. However, for the restricted cubic spine analysis, the ratios were only 4.4 and 8.8, respectively, indicating that model overfitting is possible.

Our results suggest that the relationship between insulin resistance and clinical outcomes in PD patients is complex, and differences in glycemic and lipid control alone are unlikely to account for outcome variability. Other factors, such as nutritional status and small solute clearance, may also influence clinical prognosis. Although the TyG index can serve as a metabolic health indicator, it should not replace a comprehensive risk assessment that includes inflammation markers, residual renal function, comorbidity burden, and nutritional status.

A notable strength of our study is the inclusion of both incident and maintenance dialysis patients, offering a more comprehensive view of how TyG may relate to treatment outcomes across different stages of dialysis therapy. However, being a single-center study, the generalizability of our findings may be limited. Moreover, we only measured the TyG index at baseline. In PD patients, weight, glucose exposure, lipid profile, other metabolic parameters and insulin resistance change over time. Our previous research that examined the impact of changes in the TyG index over time, however, did not find any association with clinical outcomes [20]. Because of the limitations of our database, we do not have the details of medication usage and cannot perform analysis of cause-specific hospitalization. In real life clinical practice, hospital admissions are not uncommonly the result of multiple clinical problems and it is often difficult to pinpoint a single cause of hospitalization.

## Summary

In summary, while the TyG index has shown prognostic value in some studies, its predictive validity may not be universal. Future research should involve multi-center studies and stratify participants by various factors, including parameters that assess the nutritional status of PD patients.

## Electronic supplementary material

Below is the link to the electronic supplementary material.

Supplementary Material 1

## Data Availability

The original data supporting the findings of this study are openly available in Github repository at: https://github.com/szetocc/TyG_validation/blob/main/TyG%20validation%20upload.xlsx
