# Supplementary material for: Triglyceride-glucose index and the outcome of peritoneal dialysis patients: analysis of two cohorts
Source: BMC Nephrol. 2026 Jun 10;27:480. doi: 10.1186/s12882-026-05110-w (PMC13479733; doi:10.1186/s12882-026-05110-w)
Supplement: Supplementary file 1 [file 12882_2026_5110_MOESM1_ESM.docx]

# Table S1. Correlation between TyG index and clinical parameters in incident and prevalent PD cohort

| Variable | TyG index | |
| --- | --- | --- |
| Cohort | Incident PD cohort | Prevalent PD cohort |
| Blood pressure |  |  |
| Systolic | r = -0.029, *P* = 0.635 | r = -0.107, *P* = 0.157 |
| Diastolic | r = -0.103, *P* = 0.086 | r = -0.189, *P* = 0.011 |
| Charlson’s comorbidity score | r = 0.132, *P* = 0.028 | r = 0.176, *P* = 0.019 |
| Anthropometry |  |  |
| Body weight | r = 0.122, *P* = 0.043 | r = 0.140, *P* = 0.062 |
| Body height | r = -0.081, *P* = 0.178 | r = -0.014, *P* = 0.857 |
| Body mass index | r = 0.203, *P* = 0.001 | r = 0.165, *P* = 0.028 |
| Waist circumference | r = 0.139, *P* = 0.021 | r = 0.221, *P* = 0.003 |
| Hip circumference | r = 0.028, *P* = 0.647 |  |
| SGA score | r = 0.160, *P* = 0.033 | r = 0.160, *P* = 0.033 |
| MIS score | r = -0.190, *P* = 0.012 | r = -0.190, *P* = 0.012 |
| CFS score | r = 0.076, *P* = 0.205 |  |
| Baseline blood result |  |  |
| Serum albumin | r = - 0.098, *P* = 0.121 | r = 0.033, *P* = 0.659 |
| Hemoglobin | r = -0.138, *P* = 0.029 | r = 0.033, *P* = 0.668 |
| Total cholesterol | r = 0.270, *P* < 0.001 | r = 0.106, *P* = 0.158 |
| Triglycerides | r = 0.898, *P* < 0.001 | r = 0.945, *P* < 0.001 |
| LDL cholesterol | r = 0.177, *P* = 0.003 | r = 0.281, *P <* 0.001 |
| HDL cholesterol | r = -0.462, *P* < 0.001 | r = -0.499, *P* < 0.001 |
| Fasting glucose | r = 0.582, *P* < 0.001 | r = 0.594, *P* < 0.001 |
| HbA1c | r = 0.328, *P* < 0.001 | r = 0.351, *P* < 0.001 |
| Total Kt/V | r = -0.058, *P* = 0.365 | r = -0.058, *P* = 0.447 |
| Residual GFR | r = -0.010, *P* = 0.879 | r = -0.026, *P* = 0.733 |
| NPNA | r = -0.020, *P* = 0.759 | r = -0.037, *P* = 0.626 |
| FEBM | r = 0.018, *P* = 0.779 | r = 0.065, *P* = 0.389 |
| Peritoneal transport |  |  |
| D/P4 | r = -0.075, *P* = 0.239 | r = -0.029, *P* = 0.707 |
| MTAC | r = -0.084, *P* = 0.193 | r = -0.042, *P* = 0.590 |
| Bioimpedance spectroscopy |  |  |
| Over-hydration | r = -0.015, *P* = 0.805 |  |
| E:I ratio | r = 0.085, *P* = 0.166 |  |
| Lean tissue mass | r = -0.054, *P* = 0.385 |  |
| Adipose tissue mass | r = 0.239, *P* = 0.001 |  |
| Arterial pulse wave velocity |  |  |
| brachial to ankle | r = 0.085, *P* = 0.160 |  |
| carotid to femoral | r = 0.014, *P* = 0.816 |  |
| carotid to radial | r = -0.029, *P* = 0.630 |  |

PD, peritoneal dialysis; TyG, triglyceride glucose; SGA, subjective global assessment overall score; MIS, malnutrition inflammation score; CFS, clinical frailty scale; LDL, low-density lipoprotein; HDL, high-density lipoprotein; HbA1C, glycosylated hemoglobin; Kt/V, dialysis adequacy assessment by total urea clearance; GFR, glomerular filtration rate; NPNA, normalized protein nitrogen appearance; FEBM, fat-free edema-free body mass; D/P4, dialysate-to-plasma ratios of creatinine at 4 hours; MTAC, mass transfer area coefficients; E: I ratio, extra-cellular to intracellular volume ratio.

Data are presented as correlation coefficient r and its 95% confidence interval (in the bracket). Data are presented as Spearman’s rank correlation coefficient.

# Table 3. Supplementary Cox regression model including all clinical parameters for patient survival of the incident PD cohort

|  | **Model** | **Univariate** | | **Multivariate** | | |
| --- | --- | --- | --- | --- | --- | --- |
|  | **Parameter** | **HR** | ***P*-value** | **HR** | **95% Cl** | ***P*-value** |
|  | TyG quartile | 1.263 | 0.045 | 0.958 | 0.732 to 1.123 | 0.753 |
|  | Age | 1.009 | 0.453 |  |  |  |
|  | Sex | 1.137 | 0.627 |  |  |  |
|  | Body Weight | 1.004 | 0.016 | 1.022 | 0.991 to 1.053 | 0.162 |
|  | Body mass index | 1.010 | 0.016 | 0.957 | 0.880 to 1.041 | 0.957 |
|  | Systolic blood pressure | 1.006 | 0.346 |  |  |  |
|  | Diastolic blood pressure | 0.989 | 0.274 |  |  |  |
|  | Charlson’s comorbidity score | 1.203 | 0.001 | 1.240 | 1.084 to 1.419 | 0.002 |
|  | Waist circumference | 1.010 | 0.390 |  |  |  |
|  | Hip circumference | 1.009 | 0.505 |  |  |  |
|  | Other nutritional indices |  |  |  |  |  |
|  | SGA | 0.832 | 0.144 |  |  |  |
|  | MIS | 1.128 | 0.0001 | 1.071 | 0.967 to 1.185 | 0.189 |
|  | CFS | 1.364 | 0.001 | 1.029 | 0.765 to 1.385 | 0.848 |
|  | Hemoglobin | 0.849 | 0.084 | 0.857 | 0.694 to 1.058 | 0.151 |
|  | Serum albumin | 0.926 | 0.009 | 0.983 | 0.912 to 1.059 | 0.645 |
|  | Total cholesterol | 1.045 | 0.668 |  |  |  |
|  | Triglyceride | 1.124 | 0.222 |  |  |  |
|  | LDL | 1.036 | 0.776 |  |  |  |
|  | HDL | 0.654 | 0.169 |  |  |  |
|  | Fasting glucose | 1.038 | 0.463 |  |  |  |
|  | HbA1c | 1.106 | 0.422 |  |  |  |
|  | Kt/V | 0.887 | 0.540 |  |  |  |
|  | Residual GFR | 0.948 | 0.317 |  |  |  |
|  | NPNA | 1.067 | 0.877 |  |  |  |
|  | FEBM | 1.019 | 0.172 |  |  |  |
|  | D/P4 | 1.139 | 0.774 |  |  |  |
|  | MTAC | 1.014 | 0.535 |  |  |  |
|  | Overhydration | 1.162 | < 0.0001 | 1.185 | 0.949 to 1.316 | 0.185 |
| E:I ratio | | 29.164 | < 0.0001 | 0.635 | 0.027 to 15.087 | 0.779 |
|  | Adipose tissue mass | 0.995 | 0.653 |  |  |  |
|  | Lean tissue mass | 0.999 | 0.943 |  |  |  |
|  | brachial to ankle | 1.173 | 0.005 | 1.035 | 0.886 to 1.208 | 0.667 |
|  | carotid to femoral | 0.985 | 0.509 |  |  |  |
|  | carotid to radial | 0.973 | 0.720 |  |  |  |

PD, peritoneal dialysis; TyG, triglyceride glucose; SGA, subjective global assessment overall score; MIS, malnutrition inflammation score; CFS, clinical frailty scale; LDL, low-density lipoprotein; HDL, high-density lipoprotein; HbA1C, glycosylated hemoglobin; Kt/V, dialysis adequacy assessment by total urea clearance; GFR, glomerular filtration rate; NPNA, normalized protein nitrogen appearance; FEBM, fat-free edema-free body mass; D/P4, dialysate-to-plasma ratios of creatinine at 4 hours; MTAC, mass transfer area coefficients; E: I ratio, extra-cellular to intracellular volume ratio.

Data are presented as Hazard ratio (95% confidence interval) and P value.

# Table S3. Relation between TyG index quartiles and clinical outcomes

| TyG index quartiles | Q1 | Q2 | Q3 | Q4 | *P* value |
| --- | --- | --- | --- | --- | --- |
| Incident PD cohort |  |  |  |  |  |
| Hospital admission  (per year) | 0.95 (0.00 to 11.65) | 0.90 (0.00 to 7.77) | 0.95 (0.27 to 8.96) | 1.04 (0.00 to 21.86) | 0.475 |
| Duration of hospital stay  (days per year) | 4.58 (0.00 to 205.80) | 3.14 (0.00 to 87.12) | 5.03 (0.00 to 182.50) | 7.29 (0.00 to 283.89) | 0.500 |
| Peritonitis rate  (episode per patient-year) | 0.00 (0.00 to 3.88) | 0.00 (0.00 to 1.59) | 0.00 (0.00 to 2.56) | 0.00 (0.00 to 14.04) | 0.425 |
| Prevalent PD cohort |  |  |  |  |  |
| Hospital admission  (per year) | 0.52 (0.00 to 1.41) | 0.47 (0.00 to 0.97) | 0.78 (0.27 to 1.80) | 0.54 (0.00 to 1.48) | 0.718 |
| Duration of hospital stay  (days per year) | 3.41 (0.00 to 15.54) | 1.98 (0.00 to 6.55) | 4.35 (0.00 to 18.48) | 2.82 (0.00 to 10.00) | 0.837 |
| Peritonitis rate  (episode per patient-year) | 0.11 (0.00 to 0.56) | 0.31 (0.00 to 0.54) | 0.23 (0.00 to 1.18) | 0.22 (0.00 to 0.69) | 0.602 |

PD, peritoneal dialysis; TyG index, Triglyceride-glucose index.

Data were presented as median (inter-quartile range) and compared by the Jonckheere-Terpstra test.

# Table S4. Summary of the causative organisms for the peritonitis episodes

|  | Incident PD cohort | | | | | Prevalent PD cohort | | | | |
| --- | --- | --- | --- | --- | --- | --- | --- | --- | --- | --- |
|  | Q1 | Q2 | Q3 | Q4 | all cases | Q1 | Q2 | Q3 | Q4 | all cases |
| S. aueus | 2 (4.3%) | 2 (5.1%) | 4 (10.0%) | 4 (10.8%) | 12 (7.4%) | 10 (16.1%) | 3 (5.1%) | 8 (8.4%) | 3 (5.2%) | 24 (8.8%) |
| CNSS | 2 (4.3%) | 2 (5.1%) | 2 (5.0%) | 2 (5.4%) | 8 (4.9%) | 7 (11.3%) | 8 (13.6%) | 16 (16.8%) | 9 (15.5%) | 40 (14.6%) |
| Enterococcus | 3 (6.5%) | 0 (0.0%) | 2 (5.0%) | 1 (2.7%) | 6 (3.7%) | 1 (1.6%) | 0 (0.0%) | 2 (2.1%) | 0 (0.0%) | 3 (1.1%) |
| Streptococcus | 4 (8.7%) | 6 (15.4%) | 5 (12.5%) | 2 (5.4%) | 17 (10.5%) | 1 (1.6%) | 8 (13.6%) | 11 (11.6%) | 6 (10.3%) | 26 (9.5%) |
| Corynebacterium | 0 (0.0%) | 0 (0.0%) | 0 (0.0%) | 0 (0.0%) | 0 (0.0%) | 6 (9.7%) | 1 (1.7%) | 8 (8.4%) | 3 (5.2%) | 18 (6.6%) |
| other GPS | 1 (2.2%) | 2 (5.1%) | 1 (2.5%) | 0 (0.0%) | 4 (2.5%) | 0 (0.0%) | 5 (8.5%) | 2 (2.1%) | 1 (1.7%) | 8 (2.9%) |
| Pseudomonas | 2 (4.3%) | 1 (2.6%) | 2 (5.0%) | 0 (0.0%) | 5 (3.1%) | 6 (9.7%) | 3 (5.1%) | 13 (13.7%) | 3 (5.2%) | 25 (9.1%) |
| E. coli | 4 (8.7%) | 2 (5.1%) | 4 (10.0%) | 4 (10.8%) | 14 (8.6%) | 7 (11.3%) | 7 (11.9%) | 6 (6.3%) | 7 (12.1%) | 27 (9.9%) |
| other GNB | 9 (19.6%) | 7 (17.9%) | 5 (12.5%) | 8 (21.6%) | 29 (17.9%) | 6 (9.7%) | 7 (11.9%) | 7 (7.4%) | 7 (12.1%) | 27 (9.9%) |
| Mixed growth | 3 (6.5%) | 5 (12.8%) | 4 (10.0%) | 7 (18.9%) | 19 (11.7%) | 7 (11.3%) | 7 (11.9%) | 15 (15.8%) | 14 (24.1%) | 43 (15.7%) |
| Mycobacterium | 3 (6.5%) | 1 (2.6%) | 2 (5.0%) | 2 (5.4%) | 8 (4.9%) | 3 (4.8%) | 2 (3.4%) | 0 (0.0%) | 0 (0.0%) | 5 (1.8%) |
| Fungus | 1 (2.2%) | 0 (0.0%) | 0 (0.0%) | 1 (2.7%) | 2 (1.2%) | 1 (1.6%) | 2 (3.4%) | 0 (0.0%) | 0 (0.0%) | 3 (1.1%) |
| Culture negative | 12 (26.1%) | 11 (28.2%) | 9 (22.5%) | 6 (16.2%) | 38 (23.5%) | 7 (11.3%) | 6 (10.2%) | 7 (7.4%) | 5 (8.6%) | 25 (9.1%) |
| Total | 46 | 39 | 40 | 37 | 162 | 62 | 59 | 95 | 58 | 274 |

PD, peritoneal dialysis; Q1 to Q4, quartile 1 to 4 of the triglyceride-glucose index; CNSS, coagulase negative staphylococcus species; GPS, Gram positive species; GNB, Gram negative bacteria.

# Table S5. Baseline demographic and clinical characteristics of the prevalent PD cohort

| No. of patients | 178 |
| --- | --- |
| TyG index | 9.01 ± 0.72 |
| Age (year) | 61.31 ± 10.8 |
| Sex (M:F) | 87:91 |
| Body weight (kg) | 63.5 ± 12.4 |
| Body mass index (kg/m^2^) | 24.9 ± 4.1 |
| Waist circumference (cm) | 90.6 ± 10.6 |
| Blood pressure (mmHg) |  |
| Systolic | 145.0 ± 21.9 |
| Diastolic | 75.1 ± 11.8 |
| Primary kidney disease, no. of case (%) |  |
| Diabetes mellitus | 66 (54.0%) |
| Hypertension | 17 (12.2%) |
| Glomerulonephritis | 54 (19.4%) |
| Polycystic kidney disease | 8 (3.6%) |
| Urological | 8 (3.6%) |
| Others | 6 (2.5%) |
| Unknown | 19 (4.7%) |
| Comorbidities, no. of case (%) |  |
| Diabetes Mellitus | 93 (52.2%) |
| Ischemic heart disease | 50 (28.1%) |
| Cerebrovascular accident | 53 (29.8%) |
| Peripheral vascular disease | 23 (12.9%) |
| Charlson’s comorbidity score | 6.0 ± 2.3 |
| PD specific parameters |  |
| APD, no. of case (%) | 6 (3.4%) |
| Icodextrin, no. of case (%) | 80 (44.9%) |
| exchange volume (L/day) | 6.6 ± 1.2 |
| ultrafiltration volume (L/day) | 0.84 ± 1.31 |
| glucose exposure (g/day) | 101.0 ± 17.6 |
| Other nutritional indices |  |
| SGA score | 5.4 ± 0.9 |
| MIS score | 7.3 ± 3.7 |
| Hemoglobin (g/dL) | 8.98 ± 1.53 |
| Albumin (g/L) | 34.56 ± 4.55 |
| Fasting glucose (mmol/L) | 6.11 ± 1.62 |
| HbA1C (%) | 5.14 ± 2.59 |
| Lipid profile (mmol/l) |  |
| Total Cholesterol | 4.93 ± 1.23 |
| Triglyceride | 2.12 ± 1.89 |
| LDL cholesterol | 2.90 ± 1.07 |
| HDL cholesterol | 1.29 ± 0.58 |
| Total Kt/V | 1.91 ± 0.43 |
| Residual GFR (ml/min/1.73m^2^) | 1.50 ± 2.30 |
| NPNA (g/kg/day) | 1.11 ± 0.26 |
| FEBM (kg) | 31.32 ± 8.64 |
| Peritoneal transport characteristics |  |
| D/P4 | 0.60 ± 0.14 |
| MTAC (ml/min/1.73m2) | 8.31 ± 4.46 |

PD, peritoneal dialysis; TyG, triglyceride glucose; SGA, subjective global assessment; MIS, malnutrition inflammation score; LDL, low-density lipoprotein; HDL, high-density lipoprotein; HbA1C, glycosylated hemoglobin; Kt/V, dialysis adequacy assessment by total urea clearance; GFR, glomerular filtration rate; NPNA, normalized protein nitrogen appearance, FEBM, fat-free edema-free body mass; D/P4, dialysate-to-plasma ratios of creatinine at 4 hours; MTAC, mass transfer area coefficient of creatinine.
